# 

**Published:** 1903-03-14

**Authors:** 


**CADBURY'S COCOA** ABSOLUTELY PURE.

**CADBURY'S COCOA** NO DRUGS OR ALKALIES USED.

### THE HOSPITAL

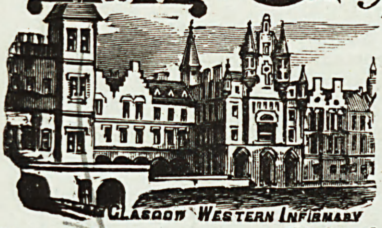

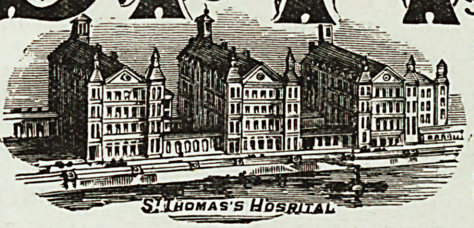

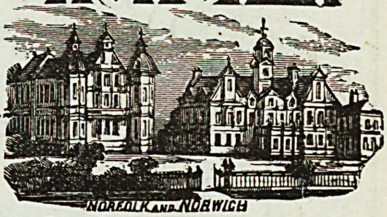

No. 859. Vol. XXXIII. [Registered as a Newspaper.] SATURDAY, MARCH 14TH, 1903. [Subscription Terms, see p. 404.] PRICE TWOPENCE.

